# Spontaneous Renal Artery Thrombosis: An Unusual Cause of Acute Abdominal Pain in the Emergency Department

**DOI:** 10.7759/cureus.43707

**Published:** 2023-08-18

**Authors:** Paul Boulos, Joe Kadou

**Affiliations:** 1 Emergency Department, Centre Hospitalier Interrégional Edith Cavell, Brussels, BEL

**Keywords:** catheter-directed thrombolysis, renal artery thrombosis, thrombosis, renal artery, renal, spontaneous, acute abdominal pain

## Abstract

Abdominal pain is a frequent complaint in the Emergency Department and thrombosis of the renal artery is an uncommon diagnosis for abdominal pain. Although the diagnosis is rare and can be difficult to make, a delayed diagnosis can lead to grave complications. This is the case of a middle-aged man who presented in the Emergency Department with left iliac fossa pain; the clinical features were not specific, and he was diagnosed with a left renal infarction. With this case, we want to remind emergency practitioners of the diagnosis and show that even late (more than six hours) thrombolysis can improve kidney perfusion and function.

## Introduction

Thrombosis of the renal artery is a rare diagnosis of abdominal pain and spontaneous thrombosis is even more uncommon, with only a few cases described in the medical literature [1-3]. The rarity of spontaneous thrombosis, the lack of specificity in the symptoms, and the non-sensitive laboratory tests make it difficult to diagnose. Although the diagnosis is rare and can be difficult to make, a delayed diagnosis can lead to grave complications (from organ loss to death). The purpose of this case is to remind emergency practitioners of the diagnosis and when to think of it as a cause of abdominal pain.

## Case presentation

A 55-year-old patient presented in the Emergency Department (ED) with severe sudden continuous pain located in the left iliac fossa that started eight hours before admission. The pain did not radiate, and the patient did not experience any lumbar discomfort. He felt feverish but did not have any fever. He did not present with any associated symptoms; in particular, there was no macroscopic hematuria or urinary or digestive problems. The patient had, however, complained of fatigue for the last 10 days. His medical history includes urolithiasis with renal colic and appendicectomy. He was not on any medication at the time and did not smoke, use drugs or drink any alcohol.

Clinical examination showed slightly elevated blood pressure (157/112 mmHg), a heart rate of 73 beats per minute, and a blood oxygen saturation level of 99%. The abdomen was tender, with pain in the left flank and left iliac fossa and a painful left lumbar shock.

A blood test was performed, and a urinary sample was sent to the laboratory. Painkillers were administered and strong doses of morphine were needed to calm the pain.

Laboratory results are shown in Table 1. In summary, we observed renal insufficiency and a slightly elevated lactate dehydrogenase (LDH) level. The urinary sample showed normal white and red blood cell counts and mild proteinuria. The electrocardiogram (ECG) showed a normal sinus rhythm.

**Table 1:** Laboratory result upon admission and after 12 hours

Biological parameters	T0	T+12hours	Normal values
C-reactive protein	14,2	23,6	<5mg/dL
Urea	37	29	18-55 mg/dL
Creatinine	1,4	1,4	<1.2 mg/dL
Platelets	141,000	113,000	150,000-400,000 /µL
Lactate dehydrogenase	262	437	<250 IU/L
Aspartate aminotransferase	36	52	<40 IU/L
Alanine transaminase	24	43	<41IU/L

An unenhanced abdominal CT scan did not show any urolithiasis and there was no sign of diverticulitis. With the high level of pain and the absence of a diagnosis, the patient was kept under surveillance in the ED. A second blood test was taken 12 hours after the first one and showed an increase in LDH, mild thrombopenia, a slight alteration of the transaminase levels, and an increasing CRP level (Table 1).

**Figure 1:**
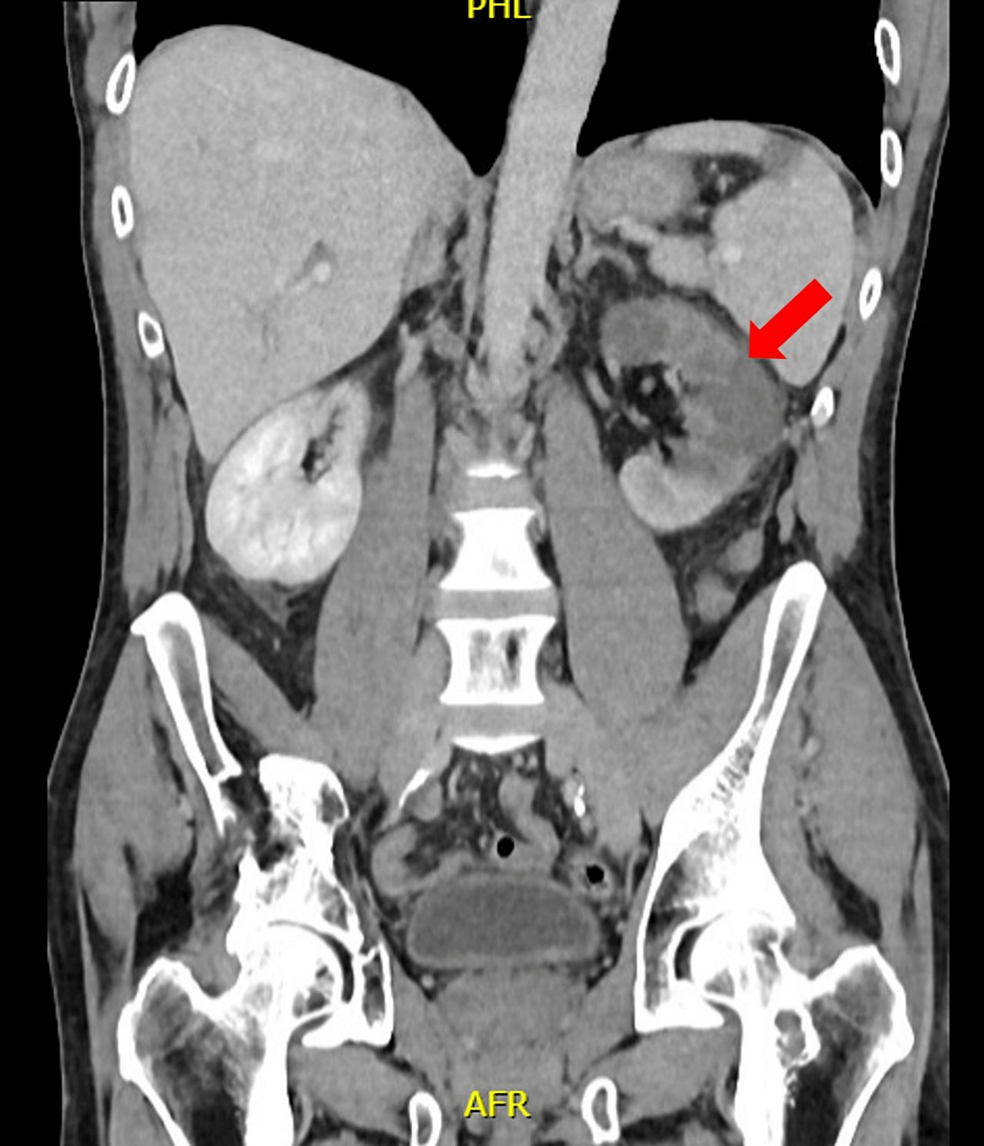
Enhanced CT scan showing a large renal infarction

**Figure 2:**
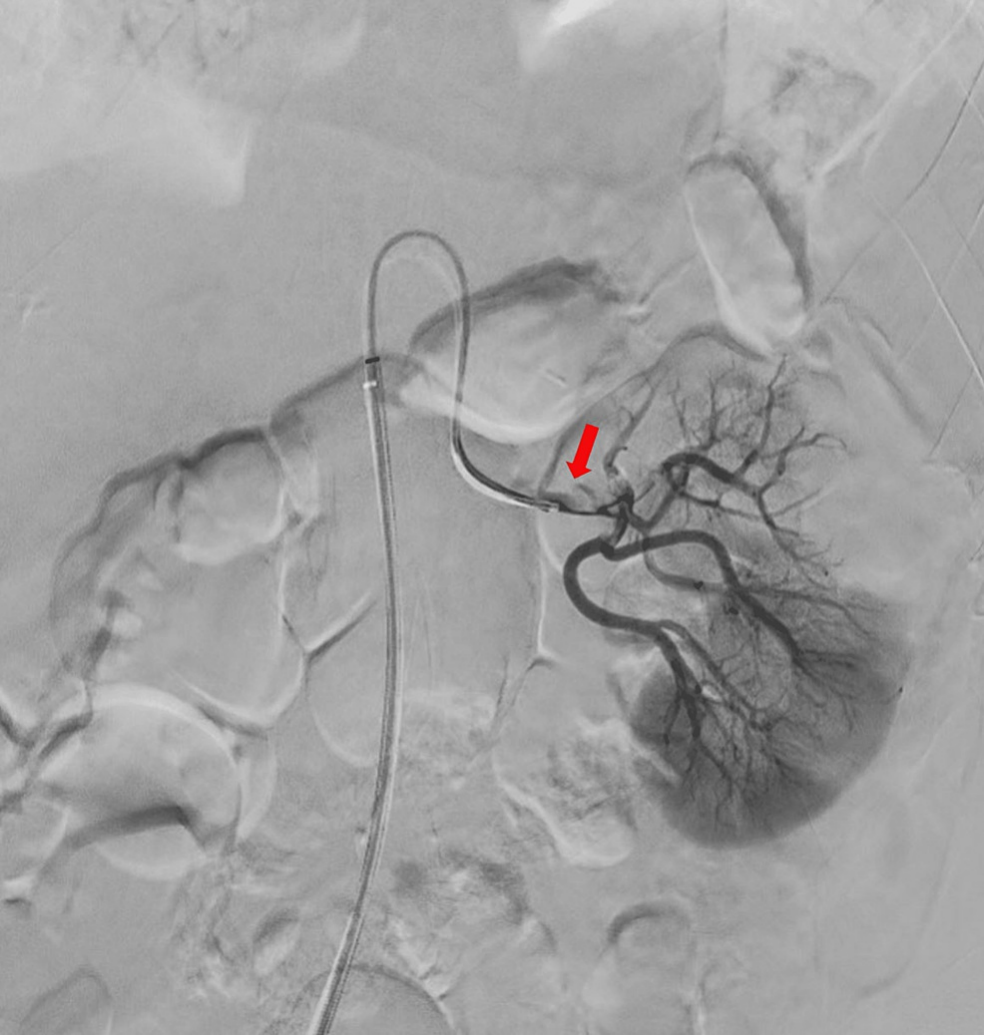
Renal angiogram showing a proximal left renal artery throbosis The red arrow shows the angiogram catheter going through the thrombus

**Figure 3:**
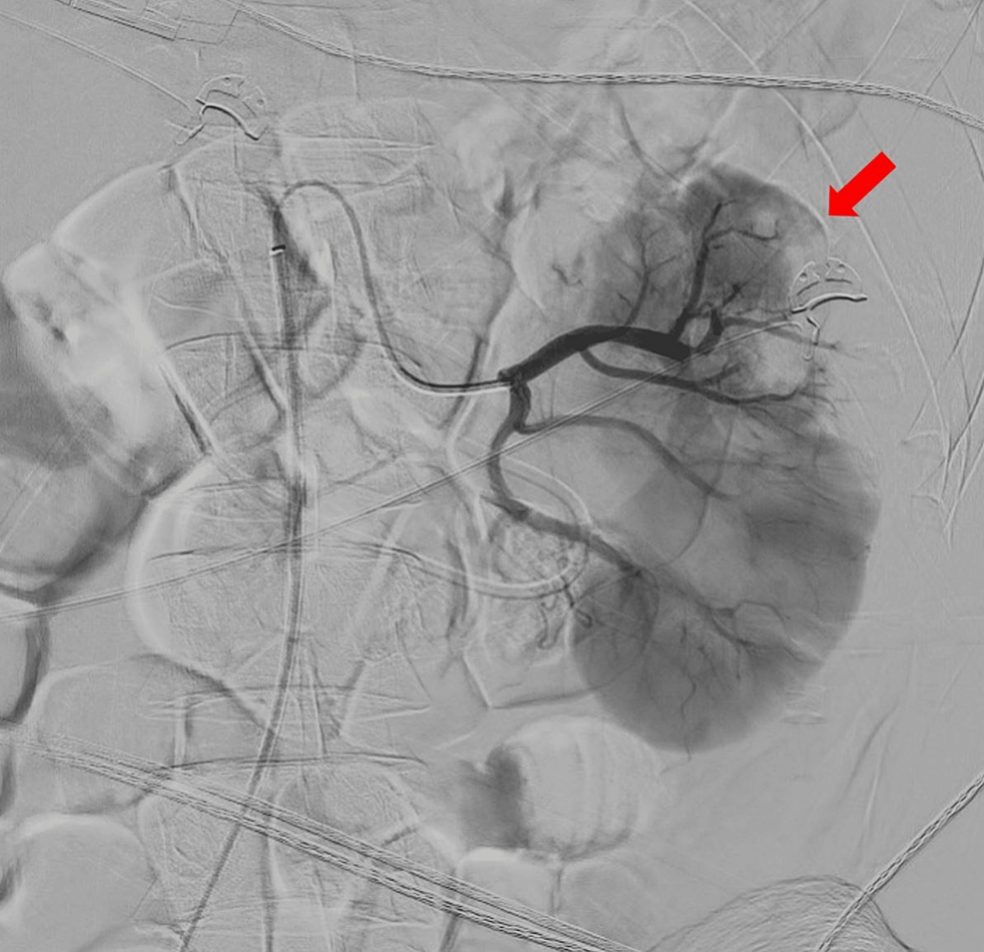
Renal angiogram after local thrombolysis showing a partial superior kidney pole reperfusion

The pain continued for the rest of the night. A contrast-enhanced abdominal scan was prescribed, and this second scan showed a left renal infarction (Figure 1). A subsequent renal angiogram showed proximal occlusion of the left renal artery, with some distal thrombi (Figure 2). During the procedure, a bolus of 100,000 IU of urokinase was administered, followed by a continuous locoregional administration of 200,000 IU per hour. The patient was then transferred to the Intensive Care Unit (ICU), where anticoagulant treatment was initiated with non-fractionated heparin. After six hours, a second angiogram showed good results, with just a few distal thrombi remaining. The dosage of urokinase was then halved. After 24 hours, the renal angiogram showed partial revascularization of the left kidney, and thrombolysis was stopped. At 48 hours, the angiogram showed partial reperfusion of the superior kidney pole (Figure 3) and the anticoagulation treatment was switched to low-molecular-weight heparin; acenocoumarol treatment was started after a few days.

A complete etiologic evaluation was carried out: the patient did not present any atrial fibrillation during monitoring in the ED, ICU, or during a later ECG Holter examination. Transthoracic and subsequent transesophageal echocardiography did not show any thrombus, embolizing origin, or valvular anomaly. Positron emission tomography did not show any sign of neoplasia. Vasculitis, autoimmune diseases, and primary causes of hyperviscosity syndromes (such as antiphospholipid syndrome, Factor V Leiden mutation, prothrombin 20110A mutation, etc.) were excluded. There was no history of blunt abdominal trauma.

Kidney function improved a little during the patient’s stay, with a final creatinine level of 1.1 mg/dL, and kidney scintigraphy showed an amputation of the upper left kidney lobe, with a global left kidney function of 21.9% (compared with 78.1% for the right kidney). The measured clearance of creatinine was 97 mL/min on day 3.

## Discussion

Spontaneous thrombosis of the renal artery is a rare condition that occurs mostly during the third to fifth decade [1,4,5]. The prevalence of renal artery thrombosis is 2/1,000,000, as shown in the largest study available [2].

The usual presentation is vague, with general symptoms of flank pain, nausea, vomiting, fever, and even diarrhea and the laboratory results lack sensitivity and can show hyperleukocytosis and high levels of LDH, which translate to a poorer prognosis [1,3-6]. A raised LDH level is usually present when renal infarction occurs; however, the values can also be normal, thus a normal value does not confirm the diagnosis [1]. The high level of LDH can contribute to the diagnosis in association with a CT scan [2,3]. Hematuria and proteinuria can also be present [1,6] and kidney function is often in the normal range [6].

Our patient presented with left iliac fossa pain, and fatigue and had a misleading history of kidney stones with renal colic. The initial laboratory results did not show raised LDH levels or any hematuria. The unenhanced scan did not show urethral lithiasis and it was only 12 hours later that LDH levels increased while the strong pain persisted, thus requiring an enhanced CT (but not specifically to rule out kidney infarction). The diagnosis was later confirmed by a renal angiogram.

In most cases of renal infarction, there is an identifiable cause, such as blunt abdominal trauma, surgery, neoplasia, dysrhythmias, thromboemboli, atheromatosis, endocarditis, and coagulopathy [1-4,6]​.​​​​​​ In our case, no identifiable cause was found but we excluded a thrombo-embolic cause (normal transesophageal echocardiography) and there was no hypercoagulability state, neoplasia, trauma, etc. Thus, the conclusion was a spontaneous or idiopathic left proximal renal artery thrombosis. A rare diagnosis with only a few cases described in the literature [1-3].

Diagnosis needs to be quick to avoid any permanent damage, but it is not easy due to the non-specificity of signs and symptoms and the lack of sensitivity in the laboratory results. The gold standard for diagnosis is an enhanced CT scan followed by a renal angiogram [3,7]. In our case, the unenhanced CT made no contribution, but the enhanced CT showed renal infarction and the angiogram showed the exact location of the thrombus. In the case of clinical suspicion of renal origin with a normal unenhanced CT, a contrast injection should complete the workup [6,7]. With our patient, the diagnosis was delayed due to the delayed enhanced CT.

The therapeutic range is vague and there are no guidelines due to the rarity of the disease [2,3]. Therapy includes thrombectomy and/or local thrombolysis, anticoagulants, and surgery [3,7]. The importance of early diagnosis is supported by the fact that the prognosis is greater if treatment starts sooner [1-3]. In some cases, thrombectomy and stenting of the renal artery were performed [4]. Thrombolysis is recommended in the first six hours (4-6 hours, depending on the published cases) [1,4]. After that period of time, thrombolysis and revascularization do not seem to significantly improve the outcome and there is a greater risk of irreversible lesions [3,4].

Our case demonstrates that even after the first six hours local thrombolysis can still improve kidney function. Locoregional thrombolysis was performed after 22 hours, and the patient's creatinine level dropped from 1.4 mg/dL to 1.1 mg/dL; the measured creatinine clearance was 97 mL/min when the patient was discharged from the ICU. With our patient, it seems that local thrombolysis can still improve kidney function and perfusion if it is achieved in the first 24 hours.

## Conclusions

Renal artery thrombosis is a rare diagnosis that needs to be rapidly diagnosed to avoid long-term complications. An enhanced CT scan should be performed when there is no explanation for the patient’s severe symptoms, contrasting with the lack of abnormalities in the laboratory and unenhanced CT scan results. Local thrombolysis represents one of the therapeutic options and is recommended in the first six hours, but in our case, we observed an improvement even when the local thrombolysis was done later.
